# Supplementary material for: Intestinal Permeability and Dysbiosis in Female Patients with Recurrent Cystitis: A Pilot Study
Source: J Pers Med. 2022 Jun 20;12(6):1005. doi: 10.3390/jpm12061005 (PMC9225239; doi:10.3390/jpm12061005)
Supplement: Supplementary file 1 [file jpm-12-01005-s001.zip › jpm-1729873-supplementary.pdf]

Gemelli

Fondazione Policlinico Universitario Agostino Gemelli IRCCS
Università Cattolica del Sacro Cuore

SUPPLEMENTARY

Questionnaire N°1:

The first questionnaire assessed whether the patient has ever had of recurrent cystitis or other urinary tract infections: how many episodes of cystitis and/or urinary tract infections occurred in the last six months or in the last year and if the patient had one or more gastrointestinal symptoms, such as: chronic inflammatory bowel diseases (Crohn's Disease or Ulcerative Colitis), positive Lactose or Lactulose Breath Test, SIBO (Small Intestinal Bacterial Overgrowth).

Gemelli

Fondazione Policlinico Universitario Agostino Gemelli IRCCS
Università Cattolica del Sacro Cuore

UOC di Medicina Interna e Gastroenterologia - Direttore Prof. A. Gasbarrini
Ambulatorio Gastroenterologia e delle Malattie Infiammatorie Croniche Intestinali
Tel. 06 30 15 62 65

Gentile paziente,

Le chiediamo di compilare il seguente questionario dove troverà domande inerenti al Suo stato di salute e/o di malattia.

Grazie per la Sua collaborazione.

Data: ____________________

Cognome: ____

Nome: ____

Data di nascita: ________________________________

Luogo di nascita: _______________________________

Sesso: M F

Etnia: ____

Impiego: ______________________________________

Recapito Telefonico: ____________________________

Mail: ____

Il sottoscritto, acquisite le informazioni di cui all'art. 13 della D. Lgs 196/2003, ai sensi dellart. 23 della legge stessa conferisce il proprio consenso al trattamento dei propri dati personali.

Firma: ______________________________________

Gemelli

Fondazione Policlinico Universitario Agostino Gemelli IRCCS
Università Cattolica del Sacro Cuore

La invitiamo, cortesemente, a rispondere alle seguenti domande:

1. ***Le è mai capitato di soffrire di cistiti e/o altre infezioni del tratto urinario?***
   - ◻ NO
   - ◻ SÌ (in caso di risposta affermativa proseguire il questionario)

2. ***In caso di risposta affermativa alla seguente domanda, indicare quanti episodi di cistite e/o infezioni del tratto urinario si sono succeduti negli ultimi sei mesi o nell'ultimo anno:***
   - ◻ Nessun episodio negli ultimi sei mesi/anno
   - ◻ Una sola volta negli ultimi sei mesi/anno
   - ◻ Due o più volte negli ultimi sei mesi
   - ◻ Due volte nell'ultimo anno
   - ◻ Tre o più volte nell'ultimo anno

3. ***Indicare se presenta una o più delle seguenti condizioni:***
   - ◻ Malattia infiammatoria cronica intestinale (M.Crohn o RettoColiteUlcerosa)
   - ◻ Positività BreathTest al Lattosio
   - ◻ Positività Breath Test al Lattulosio
   - ◻ Nessuna delle precedenti
   - ◻ Altro ____

Fondazione Policlinico Universitario Agostino Gemelli IRCCS
Università Cattolica del Sacro Cuore

Questionnaire N°2:

The second self-assessment questionnaire, ACSS / UTISA, assessed aspects of urological conditions (e.g., frequency and urgency to urinate, dysuria, inability to empty the bladder completely, pelvic pain, discomfort, macrohematuria, etc.). This questionnaire also evaluates gynecological conditions (e.g., scoring of premenstrual symptoms and presence of menopausal symptoms), pregnancy, diabetes, or history of hospital admissions (Table 2). In addition, the second questionnaire assessed the presence and severity of gastroenterological symptoms, such as: nausea, vomiting, diarrhea, abdominal pain and swelling, gastroesophageal reflux, stool frequency and consistency. Finally, the subjective perception of how these symptoms affected the patient's quality of life also considered.

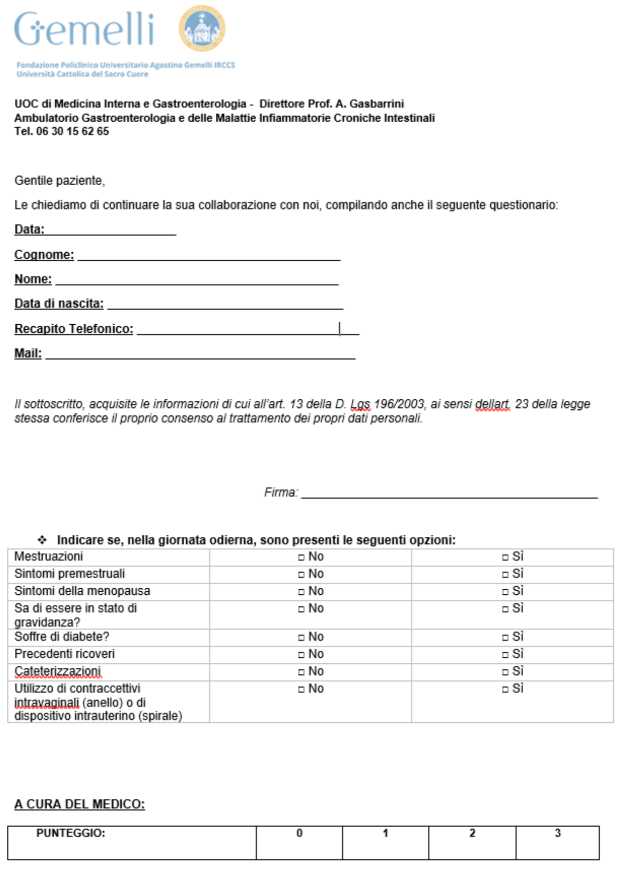

Fondazione Policlinico Universitario Agostino Gemelli IRCCS
Università Cattolica del Sacro Cuore

UOC di Medicina Interna e Gastroenterologia - Direttore Prof. A. Gasbarrini
Ambulatorio Gastroenterologia e delle Malattie Infiammatorie Croniche Intestinali
Tel. 06 30 15 62 65

Gentile paziente,

Le chiediamo di continuare la sua collaborazione con noi, compilando anche il seguente questionario:

Data: ____________________

Cognome: ____

Nome: ____

Data di nascita: ____

Recapito Telefonico: ____

Mail: ____

Il sottoscritto, acquisite le informazioni di cui all'art. 13 della D. Lgs 196/2003, ai sensi dellart. 23 della legge stessa conferisce il proprio consenso al trattamento dei propri dati personali.

Firma: ____

❖ **Indicare se, nella giornata odierna, sono presenti le seguenti opzioni:**

Mestruazioni	□ No	□ Sì
Sintomi premestruali	□ No	□ Sì
Sintomi della menopausa	□ No	□ Sì
Sa di essere in stato di gravidanza?	□ No	□ Sì
Soffre di diabete?	□ No	□ Sì
Precedenti ricoveri	□ No	□ Sì
Cateterizzazioni	□ No	□ Sì
Utilizzo di contraccettivi intravaginali (anello) o di dispositivo intrauterino (spirale)	□ No	□ Sì

A CURA DEL MEDICO:

PUNTEGGIO:	0	1	2	3

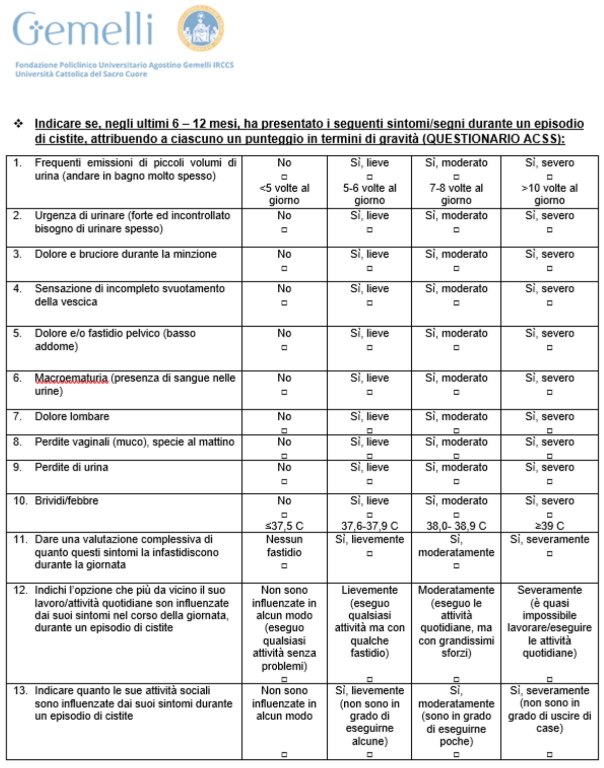

Fondazione Policlinico Universitario Agostino Gemelli IRCCS
Università Cattolica del Sacro Cuore

❖ **Indicare se, negli ultimi 6 – 12 mesi, ha presentato i seguenti sintomi/segni durante un episodio di cistite, attribuendo a ciascuno un punteggio in termini di gravità (QUESTIONARIO ACSS):**

1. Frequenti emissioni di piccoli volumi di urina (andare in bagno molto spesso)	No □ <5 volte al giorno	Sì, lieve □ 5-6 volte al giorno	Sì, moderato □ 7-8 volte al giorno	Sì, severo □ >10 volte al giorno
2. Urgenza di urinare (forte ed incontrollato bisogno di urinare spesso)	No □	Sì, lieve □	Sì, moderato □	Sì, severo □
3. Dolore e bruciore durante la minzione	No □	Sì, lieve □	Sì, moderato □	Sì, severo □
4. Sensazione di incompleto svuotamento della vescica	No □	Sì, lieve □	Sì, moderato □	Sì, severo □
5. Dolore e/o fastidio pelvico (basso addome)	No □	Sì, lieve □	Sì, moderato □	Sì, severo □
6. Macroematuria (presenza di sangue nelle urine)	No □	Sì, lieve □	Sì, moderato □	Sì, severo □
7. Dolore lombare	No □	Sì, lieve □	Sì, moderato □	Sì, severo □
8. Perdite vaginali (muco), specie al mattino	No □	Sì, lieve □	Sì, moderato □	Sì, severo □
9. Perdite di urina	No □	Sì, lieve □	Sì, moderato □	Sì, severo □
10. Brividi/febbre	No □ ≤37,5 C	Sì, lieve □ 37,6-37,9 C	Sì, moderato □ 38,0- 38,9 C	Sì, severo □ ≥39 C
11. Dare una valutazione complessiva di quanto questi sintomi la infastidiscono durante la giornata	Nessun fastidio □	Sì, lievemente □	Sì, moderatamente □	Sì, severamente □
12. Indichi l'opzione che più da vicino il suo lavoro/attività quotidiane son influenzate dai suoi sintomi nel corso della giornata, durante un episodio di cistite	Non sono influenzate in alcun modo (eseguo qualsiasi attività senza problemi) □	Lievemente (eseguo qualsiasi attività ma con qualche fastidio) □	Moderatamente (eseguo le attività quotidiane, ma con grandissimi sforzi) □	Severamente (è quasi impossibile lavorare/eseguire le attività quotidiane) □
13. Indicare quanto le sue attività sociali sono influenzate dai suoi sintomi durante un episodio di cistite	Non sono influenzate in alcun modo □	Sì, lievemente (non sono in grado di eseguirne alcune) □	Sì, moderatamente (sono in grado di eseguirne poche) □	Sì, severamente (non sono in grado di uscire di case) □

Fondazione Policlinico Universitario Agostino Gemelli IRCCS
Università Cattolica del Sacro Cuore

GSCG (Gastrointestinal Symptom Rating Scale)

Per ognuno delle 15 domande indicare un punteggio (compreso tra 0 e 3) che meglio rappresenti l'attuale severità dei sintomi percepiti (come si sente in rapporto ai Suoi disturbi al momento della visita).
Il Suo giudizio sui sintomi sarà il risultato di un bilancio dato da: intensità, frequenza, necessità di alleviarli ed impatto sociale

1) **Dolore Addominale. Esperienza soggettiva di disagio causato dai Suoi sintomi addominali e dolore. (Valutare secondo intensità, frequenza, durata, necessità di sollievo, ed impatto sociale).**
   0 dolore assente o transitorio
   1 dolori occasionali che interferiscono con le attività quotidiane
   2 dolori prolungati ed intensi. Interferiscono con molte attività e necessitano sollievo.
   3 dolori severi o paralizzanti. Interferiscono con tutte le attività sociali

2) **Bruciore di Stomaco. Sensazione di disagio causato dai Suoi sintomi addominali o bruciore in sede retrosternale. (Valutare secondo intensità, frequenza, durata, necessità di sollievo, ed impatto sociale).**
   0 nessun bruciore oppure bruciore transitorio
   1 disagio occasionale e di breve durata
   2 episodi frequenti di disagio prolungati, richiedono sollievo farmacologico
   3 disagio continuo; solo transitori benefici dagli anti-acidi

3) **Rigurgiti Acidi. Improvviso rigurgito del contenuto acido gastrico. (Valutare secondo intensità, frequenza, e necessità di sollievo).**
   0 nessun rigurgito o rigurgiti transitori
   1 rigurgiti occasionali
   2 una o due volte al giorno; necessaria assunzione farmaci
   3 rigurgiti per la maggior parte della giornata. Solo lieve sollievo da anti-acidi

4) **Sensazione Tensione alla Bocca dello Stomaco. Con sollievo dato da cibo o da anti-acidi. Se non si hanno a disposizione né cibo né anti-acidi la sintomatologia peggiora. (Valutare secondo intensità, frequenza, durata, e necessità di sollievo).**
   0 no tensione, oppure transitoria
   1 disagio occasionale e di breve durata. Non necessita di cibo o farmaci nell'intervallo tra i pasti
   2 frequenti episodi di disagio prolungato. Necessità di anti-acidi nell'intervallo tra i pasti
   3 disagio continuo. Frequente necessità di cibo o anti-acidi

5) **Nausea e Vomito. (Valutare secondo intensità, frequenza, e durata).**
   0 no nausea
   1 episodi occasionali e di breve durata
   2 nausea frequente e prolungata; no episodi di vomito
   3 nausea continua, e vomito frequente

6) **Borborigmi. Brontolio a livello addominale. (Valutare secondo intensità, frequenza, durata, ed impatto sociale).**
   0 mai o transitori
   1 borborigmi occasionali, fastidiosi ma di breve durata
   2 frequenti e prolungati episodi che possono essere gestiti senza impatto sulle attività sociali
   3 continui borborigmi che interferiscono in modo importante con l'attività quotidiana

7) **Distensione Addominale. Gonfiore a livello addominale, dovuto alla formazione di gas in eccesso. (Valutare secondo intensità, frequenza, durata, e impatto sociale).**
   0 nessuna o transitoria distensione
   1 disagio occasionale e di breve durata
   2 frequenti e prolungati episodi che possono essere gestiti regolando il capo

Fondazione Policlinico Universitario Agostino Gemelli IRCCS
Università Cattolica del Sacro Cuore

   d'abbigliamento
   3 disagio continuo che interferisce in modo importante con la vita quotidiana

8) **Eruttazioni. (Valutare secondo intensità, frequenza, ed impatto sociale).**
   0 nessuna o transitorie
   1 fastidiose eruttazioni occasionali
   2 episodi frequenti, interferenti con alcune attività sociali
   3 episodi frequenti che interferiscono seriamente con la vita sociale

9) **Flatulenza. Eccessiva espulsione di aria. (Valutare secondo intensità, frequenza, durata, ed impatto sociale).**
   0 nessun aumento della flatulenza
   1 disagio occasionale e di breve durata
   2 episodi frequenti e prolungati, interferenti con alcune attività sociali
   3 episodi frequenti che interferiscono seriamente con la vita sociale

10) **Diminuzione del numero di Evacuazioni. (Valutare solo per la frequenza con cui si verifica l'alterazione delle consistenza delle feci).**
   0 una volta al giorno
   1 ogni tre giorni
   2 ogni cinque giorni
   3 ogni sette giorni o meno frequentemente

11) **Aumento del numero di Evacuazioni. (Valutare solo per la frequenza con cui si verifica l'alterazione delle consistenza delle feci).**
   0 una volta al giorno
   1 tre volte al giorno
   2 cinque volte al giorno
   3 sette o più volte al giorno

12) **Evacuazioni Feci Liquide. (Valutare solo in base alla consistenza, indipendentemente dalla frequenza o dalla sensazione di incompleto svuotamento).**
   0 consistenza normale
   1 feci piuttosto liquide
   2 consistenza molto liquida, ma non completamente acquosa
   3 feci acquosa

13) **Evacuazione Feci Dure. (Valutare solo in base alla consistenza, indipendentemente dalla frequenza o dalla sensazione di incompleto svuotamento).**
   0 consistenza normale
   1 feci piuttosto dure
   2 feci dure
   3 feci dure e frammentate. Talvolta in combinazione con diarrea

14) **Bisogno Urgente di Defecare. Sensazione di controllo incompleto, o vera e propria incapacità di trattenere lo stimolo. (Valutare sulla base di intensità, frequenza ed impatto sociale).**
   0 controllo normale
   1 sensazione occasionale di urgente bisogno di defecare
   2 sensazione frequente di urgente bisogno di defecare, che interferisce con la vita sociale
   3 incapacità di controllare lo stimolo

15) **Sensazione di Incompleta Evacuazione. Sforzo nella defecazione, associato a sensazione di incompleta evacuazione. (Valutare in base a frequenza ed intensità).**
   0 sensazione di completa evacuazione, senza sforzo
   1 defecazione piuttosto difficile, talvolta sensazione di incompleta evacuazione
   2 defecazione decisamente difficile, spesso sensazione di incompleta evacuazione
   3 defecazione estremamente difficile, sensazione di incompleta evacuazione

Fondazione Policlinico Universitario Agostino Gemelli IRCCS
Università Cattolica del Sacro Cuore

Questionnaire N°3: The third self-assessment questionnaire is a follow-up questionnaire, which asks the patient if and by how much the uro-genito-gastroenterological symptoms reported in questionnaire N°2 improved.

Fondazione Policlinico Universitario A. Gemelli
Università Cattolica del Sacro Cuore

UOC di Medicina Interna e Gastroenterologia - Direttore Prof. A. Gasbarrini
Ambulatorio Gastroenterologia e delle Malattie Infiammatorie Croniche Intestinali
Tel. 06 30 15 62 65

Gentile paziente,

Le chiediamo di continuare la sua collaborazione con noi, compilando anche il seguente questionario:

Data: ____________________

Cognome: ________________________________

Nome: ________________________________

Data di nascita: ________________________________

Recapito Telefonico: ________________________________

Mail: ________________________________

Il sottoscritto, acquisite le informazioni di cui all'art. 13 della D. Lgs 196/2003, ai sensi dellart. 23 della legge stessa conferisce il proprio consenso al trattamento dei propri dati personali.

Firma: ________________________________

❖ **Indicare se, nella giornata odierna, sono presenti le seguenti opzioni:**

Mestruazioni	□ No	□ Sì
Sintomi premestruali	□ No	□ Sì
Sintomi della menopausa	□ No	□ Sì
Sa di essere in stato di gravidanza?	□ No	□ Sì
Soffre di diabete?	□ No	□ Sì
Precedenti ricoveri	□ No	□ Sì
Cateterizzazioni	□ No	□ Sì
Utilizzo di contraccettivi intravaginali (anello) o di dispositivo intrauterino (spirale)	□ No	□ Sì

❖ **Dall'ultima volta che ha compilato questo questionario, ci sono stati cambiamenti riguardo ai sintomi dell'infezione del tratto urinario?**
- □ No, la situazione è invariata
- □ No, la situazione è peggiorata
- □ Sì, ci sono stati miglioramenti

❖ **Se ha risposto sì alla precedente domanda, indichi quanto i suoi sintomi sono migliorati:**
- □ Migliorati molto
- □ Migliorati moderatamente
- □ Migliorati poco
- □ Migliorati a volte

Fondazione Policlinico Universitario A. Gemelli
Università Cattolica del Sacro Cuore

❖ **Indicare se, negli ultimi 6 – 12 mesi, ha presentato i seguenti sintomi/segni durante un episodio di cistite, attribuendo a ciascuno un punteggio in termini di gravità (QUESTIONARIO ACSS):**

1. Frequenti emissioni di piccoli volumi di urina (andare in bagno molto spesso)	No □ <5 volte al giorno	Sì, lieve □ 5-6 volte al giorno	Sì, moderato □ 7-8 volte al giorno	Sì, severo □ >10 volte al giorno
2. Urgenza di urinare (forte ed incontrollato bisogno di urinare spesso)	No □	Sì, lieve □	Sì, moderato □	Sì, severo □
3. Dolore e bruciore durante la minzione	No □	Sì, lieve □	Sì, moderato □	Sì, severo □
4. Sensazione di incompleto svuotamento della vescica	No □	Sì, lieve □	Sì, moderato □	Sì, severo □
5. Dolore e/o fastidio pelvico (basso addome)	No □	Sì, lieve □	Sì, moderato □	Sì, severo □
6. Macroematuria (presenza di sangue nelle urine)	No □	Sì, lieve □	Sì, moderato □	Sì, severo □
7. Dolore lombare	No □	Sì, lieve □	Sì, moderato □	Sì, severo □
8. Perdite vaginali (muco), specie al mattino	No □	Sì, lieve □	Sì, moderato □	Sì, severo □
9. Perdite di urina	No □	Sì, lieve □	Sì, moderato □	Sì, severo □
10. Brividi/febbre	No □ ≤37,5 C	Sì, lieve □ 37,6-37,9 C	Sì, moderato □ 38,0- 38,9 C	Sì, severo □ ≥39 C
11. Dare una valutazione complessiva di quanto questi sintomi la infastidiscono durante la giornata	Nessun fastidio □	Sì, lievemente □	Sì, moderatamente □	Sì, severamente □
12. Indichi l'opzione che più da vicino il suo lavoro/attività quotidiane son influenzate dai suoi sintomi nel corso della giornata, durante un episodio di cistite	Non sono influenzate in alcun modo (eseguo qualsiasi attività senza problemi) □	Lievemente (eseguo qualsiasi attività ma con qualche fastidio) □	Moderatamente (eseguo le attività quotidiane, ma con grandissimi sforzi) □	Severamente (è quasi impossibile lavorare/eseguire le attività quotidiane) □
13. Indicare quanto le sue attività sociali sono influenzate dai suoi sintomi durante un episodio di cistite	Non sono influenzate in alcun modo □	Sì, lievemente (non sono in grado di eseguirne alcune) □	Sì, moderatamente (sono in grado di eseguirne poche) □	Sì, severamente (non sono in grado di uscire di case) □

GSCG (Gastrointestinal Symptom Rating Scale)

Per ognuno dei 15 item indicare un punteggio (compreso tra 0 e 3) che meglio rappresenti **l'attuale severità dei sintomi percepiti (come si sente in rapporto ai Suoi disturbi al momento della visita).**

Il Suo giudizio sui sintomi sarà il risultato di un bilancio dato da: intensità, frequenza, necessità di alleviarli ed impatto sociale

1) **Dolore Addominale: Esperienza soggettiva di disagio causato dai Suoi sintomi addominali e dolore. (Valutare secondo intensità, frequenza, durata, necessità di sollievo, ed impatto sociale).**
   - 0 dolore assente o transitorio
   - 1 dolori occasionali che interferiscono con le attività quotidiane
   - 2 dolori prolungati ed intensi. Interferiscono con molte attività e necessitano sollievo.
   - 3 dolori severi o paralizzanti. Interferiscono con tutte le attività sociali

2) **Bruciore di Stomaco. Sensazione di disagio causato dai Suoi sintomi addominali o bruciore in sede retrosternale. (Valutare secondo intensità, frequenza, durata, necessità di sollievo, ed impatto sociale).**
   - 0 nessun bruciore oppure bruciore transitorio
   - 1 disagio occasionale e di breve durata
   - 2 episodi frequenti di disagio prolungati, richiedono sollievo farmacologico
   - 3 disagio continuo; solo transitori benefici dagli anti-acidi

3) **Rigurgiti Acidi. Improvviso rigurgito del contenuto acido gastrico. (Valutare secondo intensità, frequenza, e necessità di sollievo).**
   - 0 nessun rigurgito o rigurgiti transitori
   - 1 rigurgiti occasionali
   - 2 una o due volte al giorno; necessaria assunzione farmaci
   - 3 rigurgiti per la maggior parte della giornata. Solo lieve sollievo da anti-acidi

4) **Sensazione Tensione alla Bocca dello Stomaco: Con sollievo dato da cibo o da anti-acidi. Se non si hanno a disposizione né cibo né anti-acidi la sintomatologia peggiora. (Valutare secondo intensità, frequenza, durata, e necessità di sollievo).**
   - 0 no tensione, oppure transitoria
   - 1 disagio occasionale e di breve durata. Non necessita di cibo o farmaci nell'intervallo tra i pasti
   - 2 frequenti episodi di disagio prolungato. Necessità di anti-acidi nell'intervallo tra i pasti
   - 3 disagio continuo. Frequente necessità di cibo o anti-acidi

5) **Nausea e Vomito: (Valutare secondo intensità, frequenza, e durata).**
   - 0 no nausea
   - 1 episodi occasionali e di breve durata
   - 2 nausea frequente e prolungata; no episodi di vomito
   - 3 nausea continua, e vomito frequente

6) **Borborigmi: Brontolio a livello addominale. (Valutare secondo intensità, frequenza, durata, ed impatto sociale).**
   - 0 mai o transitori
   - 1 borborigmi occasionali, fastidiosi ma di breve durata
   - 2 frequenti e prolungati episodi che possono essere gestiti senza impatto sulle attività sociali
   - 3 continui borborigmi che interferiscono in modo importante con l'attività quotidiana

Fondazione Policlinico Universitario A. Gemelli
Università Cattolica del Sacro Cuore

7) **Distensione Addominale: Gonfiore a livello addominale, dovuto alla formazione di gas in eccesso. (Valutare secondo intensità, frequenza, durata, e impatto sociale).**
   - 0 nessuna o transitoria distensione
   - 1 disagio occasionale e di breve durata
   - 2 frequenti e prolungati episodi che possono essere gestiti regolando il capo d'abbigliamento
   - 3 disagio continuo che interferisce in modo importante con la vita quotidiana

8) **Eruttazioni.** (Valutare secondo intensità, frequenza, ed impatto sociale).
   - 0 nessuna o transitorie
   - 1 fastidiose eruttazioni occasionali
   - 2 episodi frequenti, interferenti con alcune attività sociali
   - 3 episodi frequenti che interferiscono seriamente con la vita sociale

9) **Flatulenza: Eccessiva espulsione di aria. (Valutare secondo intensità, frequenza, durata, ed impatto sociale).**
   - 0 nessun aumento della flatulenza
   - 1 disagio occasionale e di breve durata
   - 2 episodi frequenti e prolungati, interferenti con alcune attività sociali
   - 3 episodi frequenti che interferiscono seriamente con la vita sociale

10) **Diminuzione del numero di Evacuazioni. (Valutare solo per la frequenza con cui si verifica l'alterazione delle consistenza delle feci).**
   - 0 una volta al giorno
   - 1 ogni tre giorni
   - 2 ogni cinque giorni
   - 3 ogni sette giorni o meno frequentemente

11) **Aumento del numero di Evacuazioni. (Valutare solo per la frequenza con cui si verifica l'alterazione delle consistenza delle feci).**
   - 0 una volta al giorno
   - 1 tre volte al giorno
   - 2 cinque volte al giorno
   - 3 sette o più volte al giorno

12) **Evacuazioni Feci Liquide. (Valutare solo in base alla consistenza, indipendentemente dalla frequenza o dalla sensazione di incompleto svuotamento).**
   - 0 consistenza normale
   - 1 feci piuttosto liquide
   - 2 consistenza molto liquida, ma non completamente acquosa
   - 3 feci acquosa

13) **Evacuazione Feci Dure. (Valutare solo in base alla consistenza, indipendentemente dalla frequenza o dalla sensazione di incompleto svuotamento).**
   - 0 consistenza normale
   - 1 feci piuttosto dure

Fondazione Policlinico Universitario A. Gemelli
Università Cattolica del Sacro Cuore

2 feci dure

3 feci dure e frammentate. Talvolta in combinazione con diarrea

14) **Bisogno Urgente di Defecare. Sensazione di controllo incompleto, o vera e propria incapacità di trattenere lo stimolo. (Valutare sulla base di intensità, frequenza ed impatto sociale).**

0 controllo normale

1 sensazione occasionale di urgente bisogno di defecare

2 sensazione frequente di urgente bisogno di defecare, che interferisce con la vita sociale

3 incapacità di controllare lo stimolo

15) **Sensazione di Incompleta Evacuazione. Sforzo nella defecazione, associato a sensazione di incompleta evacuazione. (Valutare in base a frequenza ed intensità).**

0 sensazione di completa evacuazione, senza sforzo

1 defecazione piuttosto difficile, talvolta sensazione di incompleta evacuazione

2 defecazione decisamente difficile, spesso sensazione di incompleta evacuazione

3 defecazione estremamente difficile, sensazione di incompleta evacuazione
